# “To fast or not to fast?” Ramadan and religiosity through the eyes of people with bipolar disorder: an exploratory study

**DOI:** 10.3389/fpsyt.2023.1270000

**Published:** 2023-10-16

**Authors:** Imen Mejri, Uta Ouali, Petra C. Gronholm, Yosra Zgueb, Abdelhafidh Ouertani, Fethi Nacef

**Affiliations:** ^1^Department Psychiatry A, Razi Hospital La Manouba, Tunis, Tunisia; ^2^Faculty of Medicine of Tunis, University of Tunis El Manar, Tunis, Tunisia; ^3^Centre for Global Mental Health and Centre for Implementation Science, Institute of Psychiatry, Psychology and Neuroscience, King's College London, London, United Kingdom

**Keywords:** bipolar disorder, fasting, Ramadan, religion, stigmatization

## Abstract

**Background:**

The month of Ramadan, due to its changes in social rhythms, can seriously affect the course of bipolar disorder (BD). Therefore, psychiatrists sometimes find it necessary to discourage Ramadan practices, especially fasting, although taking part in this practice can give a sense of belonging and accomplishment to patients. Research on this subject is limited.

**Aim:**

The aim of the present work was to explore: (i) religious practices with special attention to Ramadan before and after the onset of BD, (ii) the perceptions and behaviors related to not fasting during Ramadan in patients with BD and their families’ attitudes, (iii) religiosity and self-stigmatization and their relationships with religious practices, and (iv) the doctor-patient relationship around fasting.

**Methods:**

We conducted a retrospective, cross-sectional and descriptive study in clinically stabilized patients with BD in a public mental hospital and in a private psychiatric practice in Tunis, Tunisia. Socio-demographic and clinical data, as well as data related to general religious practices and Ramadan practices were collected using a self-established questionnaire. We assessed (i) religiosity of the patients with the Duke University Religion Index and (ii) self-stigma using the Internalized Stigma of Mental Illness scale.

**Results:**

Our sample consisted of 118 patients of whom 65.3% were fasting regularly before BD onset. More than half had stopped this practice following BD onset. Of the patients who did not fast, 16% felt guilty about this and 4.9% reported receiving negative remarks from their surroundings. High self-stigma scores were observed in 11% of the patients. Self-stigma was associated significantly with negative perception of not fasting, negative remarks regarding not fasting and taking both meals at regular times during Ramadan. The decision whether to fast or not was taken without seeking medical advice in 71.2% of the sample, and 16.9% of the sample reported that their psychiatrist had spontaneously approached the issue of Ramadan fasting.

**Conclusion:**

Religiosity and more specifically the practice of Ramadan remains an important point that should be considered when treating patients with psychiatric problems. It seems necessary that healthcare professionals should integrate the positive and the negative side of fasting into their reflections. Our results remain exploratory and encourage further work on the subject.

## 1. Introduction

Bipolar disorder (BD) is a recurrent and frequent pathology that affects 1% of the general population with variations of 0.7 to 1.7% in international studies (1, 2). Etiology of BD involves complex interactions between genetic, biological, chronobiological, psychological and environmental factors (3, 4).

Many studies have focused on the relationship between BD and social rhythm (5). The social rhythm refers to the day-to-day variability of daily, habitual behaviors. As it is an external synchronizer of our biological clock, it plays an important role in the resetting phase of the circadian rhythms. Thus, life events are at the origin of the disruption of social rhythms, themselves generators of disturbance of biological rhythms which are involved in the emergence of mood episodes (6).

Islam is the most prevalent religion in Tunisia with an estimated 99% of Tunisia’s inhabitants being Sunni Muslims. The so-called “five pillars” constitute the basic norms of Islamic practice and are accepted by Muslims globally. They include prayers and fasting.

Ramadan is the ninth lunar month in the Hijri calendar. The 11-day difference from the solar calendar causes the lunar months to cycle through all the seasons and to have a variable duration between dawn to sunset depending on the geographic location and the season in which they occur.

Fasting during Ramadan is prescribed for all pubescent Muslims in good mental and physical health. During this month, the individual abstains from the oral intake of any liquid or solid substance (including medication), smoking and having sexual activities between dawn and sunset. Spiritual demands are also required such as refraining from committing any vice.

The month of Ramadan is characterized by a total disruption of the organization of daily life, including the quality and timing of meals and drug intake, the duration of sleep, the duration and schedule of work, as well as routine activities. There is almost a reversal of the activities characterizing the day and the night. Besides, the obligation of fasting during the day can compromise therapeutic compliance (7). Given the impact of Ramadan on chronobiology, it can sometimes be necessary to discourage this practice in patients living with BD.

Published literature on BD and Ramadan is scant (5) and, to date, its reporting does not allow to conclude on a consensus on the impact of the month of Ramadan on patients with BD. Within the limited available research on Ramadan and its relationship to BD, most studies focus on the clinical impact of the changes in rhythm caused by the month of Ramadan in patients with BD and do not evaluate the cultural and religious practices of the patients and their experiences during the month.

The majority of these studies were conducted in countries with a Muslim majority (such as Morocco, Iran and Pakistan), and report somewhat contradictory results. Three Moroccan studies found relapses in previously stable patients during the month of Ramadan: a prospective study (8) observed an increase of relapses during Ramadan in 45% of the cases (77.7% were manic relapses), despite the stabilization in the blood rates of lithium. Another prospective study (9) comparing two groups of fasting and not fasting patients with BD, reported that fasting during the month of Ramadan increased the risk of relapse by 2.77 amongst patients with BD (33.3% relapses were was observed during Ramadan). A third study (10), exploring the impact of fasting on plasma levels of mood stabilizers and on sleep patterns, found that 44% of relapses occurred during Ramadan (53.5% depressive relapses).

On the contrary, two other studies have shown improvement in mania and depression scores: a study including 62 Pakistani patients with BD (11) concluded that there was no significant difference in adverse events related to lithium and that depression and manic symptoms improved during and after Ramadan. A retrospective study in Iran (12) aiming to evaluate the prevalence of BD among inpatients in the month of Ramadan in comparison with other lunar months found that more hospitalizations happened in the month following Ramadan.

Advising patients with BD on Ramadan fasting remains difficult compared to, for example, diabetic patients (13, 14). However, providing such guidance would be important, as participating in Ramadan fasting can give a person a sense of belonging, which is important for this group of patients that is often marginalized by society (15, 16). On the contrary, not taking part in this religious practice can cause stigma, guilt and exclusion: literature shows that stigma is a major concern for people with BD, with a significant impact on social support, functioning, quality of life (17, 18) and self-esteem (19). Studies have found that this feeling of discrimination is much higher in patients with BD than in those with somatic illnesses and that the stigma surrounding BD is different from that of unipolar depression (20).

A number of studies have suggested that religious beliefs and practices are helpful in dealing with the extreme stress that mental illness can cause (21) by promoting a sense of well-being and helping to accept and control negative emotions (22). Religious group activities, as practiced during the month of Ramadan, seem particularly beneficial for the well-being of patients living with psychiatric disorders (23). Thus, patients and doctors may have very different expectations and perceptions about the necessity to practice Ramadan.

To the best of our knowledge, no studies have explored the perceptions and behaviors of patients in relation to fasting and other religious practices during Ramadan. Therefore, the current study is an initial exploration of an area where prior research is limited. The aims of our work were (i) to study religious practices with special attention to Ramadan before and after the onset of BD, (ii) to study the perceptions and behaviors related to not fasting during Ramadan in patients with BD and their families’ attitudes, (iii) to assess religiosity and self-stigma and their relationships with religious practices, and (iv) to explore the doctor-patient relationship around fasting.

## 2. Materials and methods

### 2.1. Study design and participants

We conducted a retrospective, cross-sectional and descriptive study during a period of 8 months (from June 2018 to January 2019).

Study participants were recruited from three psychiatric departments of Razi Hospital La Manouba, a tertiary care psychiatric specialty hospital in Tunis/Tunisia and from one private psychiatric practice. We chose to recruit from different locations to ensure diversity in terms of socio-demographic background (rural or urban residence, educational and socio-economic level) and in terms of severity of the BD.

Patients were eligible for the study if they were aged ≥18 years, were of Muslim religion, had a diagnosis of BD according to the Diagnostic and Statistical Manual of Mental Disorders, fifth edition, (DSM5) (24) had been followed for the disorder for ≥1 year, had been stable for at least 3 months prior to study intake with Hamilton Depression Rating Scale (HDRS) (25) score < 8; Young Mania Rating Scale (YMRS) (26) score ≤ 12, and gave free and informed consent to take part in this study. Patients with intellectual disability, cognitive impairment or irregular medical follow-up during the 3 months preceding the study were excluded.

### 2.2. Measures

For the purpose of this study, we developed a structured questionnaire containing close-ended questions with some nested open-ended questions in order to allow in-depth understanding of key domains of interest to this study.

The questionnaire consisted of five parts exploring different items as follows: (1) **sociodemographic** variables (age, sex, level of education, occupation, marital status, the socio-economic level). (2) **medical history** (family psychiatric history, personal health history, personal history of attempted suicide, the use of tobacco and other substances); (3) **clinical** variables (the type of BD, duration of follow-up, number and nature of relapses, comorbidities and treatment prescribed); (4) **religious practices of the patient** before and after the onset of the disorder (fasting, prayer, and visits of the mosque); (5) variables related to **patients’ perceptions and behavior during the month of Ramadan** (reasons for not fasting (own choice, advice from the attending physician or by influence of the media). In case the patient did not fast, his or her perceptions and behaviors towards not fasting were recorded, and we asked for the **families’ attitudes** towards the non-fasting patient. Last, the (6) variables reflecting **doctor-patient relationship around fasting** (treatment compliance, decision making around fasting, type of advice from attending psychiatrist) were recorded.

All items related to domain (5) (perceptions and behaviors during Ramadan and family attitudes) were informed through previously conducted formative work (qualitative interviews with 10 individuals with BD) which yielded themes related to indecisiveness whether to fast or not, difficulties with medication compliance, arrangements within the family, feelings of shame or marginalization for not fasting.

**Internalized stigma** was assessed using the brief version of The Internalized Stigma of Mental Illness (Brief ISMI) scale (27), adapted from the original ISMI scale developed by Boyd et al. (28).

The Brief ISMI is a 10-item self-report questionnaire assessing the main domains of self-stigma: alienation, stereotype endorsement, experience of discrimination, social withdrawal and stigma resistance. Each item is rated on a 4-point Likert scale: from 1 (strongly disagree) to 4 (strongly agree). Higher total scores are indicative of higher levels of reported internalized stigma. Score interpretation divides the patients into two groups: 1.00–2.50: do not report high internalized stigma, 2.51–4.00: report high internalized stigma. We used a version translated and adapted to the Tunisian dialect as part of the INDIGO-Depression study (29).

**Religiosity** was assessed using the Duke University Religion Index (DUREL) (30), which is a five-item measure of religious involvement. The instrument includes subscales assessing the three major dimensions of religiosity: (1) organizational religious activity (ORA), which involves public religious activities such as participation in group religious practices (prayer groups, religious conferences, etc.), (2) non-organizational religious activity (NORA), which includes religious activities carried out in private (saying prayers, following religious programs on TV or the radio, etc.), and (3) intrinsic religiosity (IR), which assesses the degree of personal religious commitment or motivation.

The first subscale corresponds to the first question in the DUREL and asks about frequency of attendance at religious services (ORA). The second subscale corresponds to the second question and asks about frequency of private religious activities (NORA). The third subscale consists of the final three items that assess subjective religiosity (IR). Each subscale score is examined independently: each of the first two subscales is rated on a 6-point Likert scale: from 1 (never) to 6 (more than once in a week/day). Each item of the third subscale is rated on a 5-point Likert scale: from 1 (strongly disagree) to 5 (strongly agree). We used a version that we translated into Tunisian Arabic dialect.

### 2.3. Procedure

Data were collected through structured patient interviews after their regular consultations and supplemented with information from medical records. After receiving informed consent from the study participant, we administered the depression (HDRS) and mania (YMRS) scales to check the absence of any active mood symptoms. Subsequently, study participants completed the structured interview with the self-established questionnaire and completed the measures for internalized stigma and religiosity. The interview procedure lasted about 30 to 45 min and was conducted by one investigator (IM).

### 2.4. Data analyses

Statistical analysis was performed using IBM® SPSS® statistics (version 20.0).

Descriptive statistics were made using means, medians and extremes. Comparisons were made using the Chi-square test and Fisher’s exact test for continuous variables. For quantitative variables, comparisons were made by the student’s *t*-test and, in case of non applicability (the distribution being non-normal), by the Mann–Whitney U-test. The Kruskal Wallis Test was used in case of three or more categorical, independent groups. The level of significance was set at a probability *p* < 0.05.

A number of descriptive and analytic explorations were conducted to address the study aims. Specifically, this involved:

Study aim (i) “religious practices with special attention to Ramadan before and after the onset of BD” was assessed through descriptive analyses reporting frequencies for variables capturing patient religious practices relating to (1) fasting, (2) prayers, and (3) visits to the mosque before and after BD onset.

Study aim (ii) “perceptions and behaviors related to fasting during Ramadan in patients with BD and their families” attitudes’ were likewise explored through reporting descriptive frequencies of key variables (e.g., type of continued fasting, negative feelings regarding not fasting, family attitudes towards their non-fasting family member such as acceptance, compassion and indifference).

Study aim (iii) “religiosity, self-stigma and their relationships with religious practices” was assessed through descriptive and analytic methods. The relationship between levels of religiosity (using the three different scores of the DUREL) and religious practices was explored using the Mann–Whitney U test and the Kruskal Wallis test. Each of the DUREL domains (ORA/NORA/IR) was associated to the discontinuation of the religious practices after BD onset: (1) fasting, (2) prayers, (3) visits of the mosque as well as (4) the negative perception of not fasting. In addition, ORA, NORA, and IR domains were associated to (5) fasting. The relationship between levels of self-stigma (established by the ISMI-scale) and religious practices was tested using the Pearson chi square test or Fischer’s exact test and the Kruskal Wallis test. ISMI scores were correlated to the discontinuation of religious practices after BD onset: (1) fasting, (2) prayers, (3) visits to the mosque, as well as (4) the fact of taking both meals at regular times during Ramadan, (5) a negative perception of not fasting, (6) negative remarks by the family about not fasting, and (7) fasting during Ramadan.

Study aim (iv) “doctor-patient relationship around fasting” were also addressed through descriptive analyses, reporting frequencies of variables in focus (e.g., seeking medical advice for fasting, continuing regular medication during Ramadan, being approached by psychiatrist with advice vs. seeking advice from psychiatrist regarding Ramadan fasting, and whether this advice was followed).

### 2.5. Ethical considerations

Ethical approval was obtained from Razi Hospital Ethics committee (reference: RPA2/2018). Prior to study intake, all participants provided informed consent. The study was conducted in accordance with the Declaration of Helsinki.

## 3. Results

### 3.1. Sample and sociodemographic characteristics

Our sample consisted of 118 patients. The socio-demographic and clinical characteristics of our population are summed in Table 1.

**Table 1:** Socio-demographic and clinical characteristics of the sample (*n* = 118).

**Characteristics**	**Categorical data: *n* (%)**	**Continuous data**
**Demographic characteristics**		
Sex		
Male	68 (57.6%)	
Female	50 (42.4%)	
Age, years (mean)		41 ± 11,6
Level of education		
No formal education	9 (7.6%)	
Primary education	42 (35.6%)	
Secondary education	45 (38.1%)	
Higher education	22 (18.6%)	
Professional activity		
Regular professional activity	49 (41.5%)	
Irregular professional activity	20 (16.9%)	
No professional activity	27 (22.9%)	
Works at the home	20 (16.9%)	
Retired	2 (1.7%)	
Marital status		
Single	64 (54.2%)	
Married	45 (38.1%)	
Divorced	8 (6.8%)	
Widowed	1 (0.8%)	
**Clinical characteristics**		
Diagnosis		
BD type I	108 (91.5%)	
BD type II	10 (8.5%)	
Age at the onset of the disorder, years (mean)		27,8 ± 10
Duration of follow-up, years (mean)		13,3 ± 9,3
Treatment prescribed		
Mood stabilizers	116 (98.3%)	
Antipsychotics	91 (77.1%)	
Combination of antipsychotics and mood stabilizers	88 (74.6%)	
Antidepressant	10 (8.5%)	
Benzodiazepine	50 (42.4%)	
Anticholinergic antiparkinsonian agents	26 (22%)	
Number of hospitalizations (median)		3.54
Number of manic relapses (median)		3.45
Number of depressive relapses (median)		0.55

### 3.2. Descriptive analyses addressing study aims

#### 3.2.1. Religious practices with special attention to Ramadan before and after the onset of BD

##### 3.2.1.1. Fasting

Nearly two thirds (65.3%) of patients (*n* = 77) were fasting regularly before BD onset. More than half of them had stopped this practice after BD onset. The main reasons reported by the patients for quitting fasting were: to ensure a therapeutic compliance (62.7%), the inability to stop smoking (9.8%), excessive fatigue induced by fasting (7.8%) and coexistence of a physical illness (5.9%). Other reasons were also mentioned such as: increased appetite due to antipsychotic medication, increased feeling of stress caused by fasting, fear of relapses, and Trihexyphenidyl dependence. Almost half of the patients (45%) observed fasting during Ramadan after BD onset (31.4% continued to fast regularly and 13.6% fasted irregularly).

##### 3.2.1.2. Prayers

More than a third (36.4%) of patients (*n* = 43) prayed regularly before BD onset with an average age at the start of this religious practice of 15.4 ± 4.8 years. Among the patients who prayed regularly before the onset of BD, 34.9% (*n* = 15) had stopped doing this practice following the onset of the psychiatric disorder.

Changes in the performance of this religious practice were noted in both directions in 34 patients; 14.4% of the sample (*n* = 17) stopped praying after the onset of BD, the reasons given were mainly: apragmatism, lack of concentration, asthenia, feeling of guilt about not observing all the recommendations of the Coran, and limb tremor.

Meanwhile, 14.4% (*n* = 17) started praying after BD onset mainly by own conviction or for its anxiolytic effect.

##### 3.2.1.3. Visits of the mosque

About a third (33.1%) of patients (*n* = 39) attended the mosque before BD onset. Among them, 30.8% (*n* = 12) had stopped this practice following the onset of the psychiatric disorder for different reasons such as ideas of reference, intolerance to the crowd, no beneficial effect, fatigue, feeling of guilt.

Nine patients (7.6%) began to attend the mosque after BD onset.

The reasons for of this change were essentially the own conviction (coming closer to God) for four patients, the anxiolytic effect of prayer for three patients and the mystical ideas accompanying relapses for two patients.

#### 3.2.2. Perceptions and behaviors related to fasting during Ramadan in patients with BD and their families’ attitudes and behaviors

In our sample, 81 participants (68.7%) did not fast or fasted irregularly after BD onset. Of these: 82.7% continued to have their two other meals (breakfast and lunch) at their usual times, 21% ate, drank and smoked hidden from their family, 16% felt bad about not fasting (shame, regret, isolation, culpability).

Attitudes and behavior of the family are presented in Table 2.

**Table 2:** Families’ attitudes and behavior towards their non-fasting family member (*n* = 81).

**Attitudes and behavior of the family**	***n* (%)**
Acceptance	72 (88.9%)
Compassion	1 (1.2%)
Accusation and devaluation	3 (3.7%)
Indifference	3 (3.7%)
Consider it a sin	2 (2.5%)
Make explicit negative remarks	4 (4.9%)

#### 3.2.3. Religiosity, self-stigma, and their relationships with religious practices

##### 3.2.3.1. Religiosity and its relationship with religious practices

The total Organizational Religious Activities (ORA) domain (involving public religious activities) score ranged from 1 to 6 with a mean of 2.61 and a standard deviation of 1.79.

The Total Non-Organizational Religious Activities (NORA) domain (including religious activities carried out in private) score ranged from 1 to 6 with a mean of 2.44 and a standard deviation of 1.63.

The total intrinsic religiosity (IR) domain (exploring the subjective religiosity) score ranged from 8 to 15 with an average of 11.47 and a standard deviation of 1.69.

The relationship between the DUREL’s different domains and religious practices is shown in Table 3.

**Table 3:** Association between DUREL’s different domains and religious practices.

	**Value**	**Z**	*df*	**Chi square**	***p*-value**
ORA/Discontinuation of fasting	519.500	−2.192.			0.028
NORA/Discontinuation of fasting	561.000	−1.749			0.080
IR/Discontinuation of fasting	621.500	−1.111			0.267
ORA/Discontinuation of prayers	186.500	−0.610			0.542
NORA/ Discontinuation of prayers	123.500	−2.268			0.023
IR/Discontinuation of prayers	192.000	−0.492			0.623
ORA/Discontinuation of going to mosque	119.500	−1.319			0.187
NORA/Discontinuation of going to mosque	123.000	−1.223			0.221
IR/Discontinuation of going to mosque	160.000	−0.065			0.948
ORA/Negative perception of not fasting			4	4.021	0.403
NORA/Negative perception of not fasting			4	6.294	0.178
IR/Negative perception of not fasting			4	8.135	0.087
ORA/Fasting			2	13.990	0.001
NORA/Fasting			2	13.472	0.001
IR/Fasting			2	9.962	0.007

The ORA domain was inversely proportional to the cessation of fasting after BD onset with a statistically significant association (*p* = 0.028), i.e., patients with high ORA scores were the least likely to stop the practice of fasting after the onset of BD.

The religiosity score (in its three domains explored separately) was higher when the patient chose to fast during the month of Ramadan. This association was statistically significant (*p* = 0.001).

##### 3.2.3.2. Self-stigma and its relationship with religious practices

The mean total self-stigma score was 1.85 and ranged from 1 to 3.1. We observed high self-stigma scores in 11% of the patients. Self-stigma was significantly associated significantly with negative perception of not fasting, negative remarks regarding not fasting and taking both meals at regular times during Ramadan (Table 4).

**Table 4:** Association between ISMI total score and religious practices.

	*p*-value	Exact sig (1-sided)	Chi square
ISMI/Discontinuation of fasting	0.454	0.246	
ISMI/Discontinuation of prayers	0.043	0.043	
ISMI/Discontinuation of going to mosque	0.573	0.360	
ISMI/Taking both meals at regular times during Ramadan	0.004	0.004	
ISMI/Negative perception of not fasting	0.022		11.472
ISMI/Negative remarks about not fasting	0.004	0.044	
ISMI/Fasting	0.64		5.508

#### 3.2.4. Doctor-patient relationship around fasting

The decision whether to fast or not was taken without seeking medical advice in 71.2% of the sample either by own conviction or following the advice of an Imam.

The majority of the patients who fasted stated that they took their medication regularly during Ramadan, 81.1% adapted the schedules for taking treatment according to the fasting: medication administration was divided into 2 daily doses at Suhoor (meal just before dawn) and Iftar (fast-breaking).

Twenty patients (16.9% of the sample) reported that their psychiatrist had spontaneously approached the issue of Ramadan fasting. In contrast, 27.1% of patients (*n* = 32) said that they were the first to seek the advice of their treating psychiatrist on this subject. Only two among these patients (6.2%) had not followed the advice given to them by the psychiatrist because they believed that “fasting is sacred.” For the rest of the patients (*n* = 66), the main reason for not asking the psychiatrist was that they considered fasting to be a personal choice.

When the psychiatrist gave his opinion, he advised not to fast in 50% of cases and being able to fast in 9.6% of cases (*n* = 5). In 40.4% of cases (*n* = 21), the psychiatrist did not give any specific advice and recommended to the patient to either base his judgment on his own past experiences from the previous Ramadan months or to try to fast for a few days and then decide whether to continue or not. According to the patients, in 48% of the cases, the psychiatrist had given arguments explaining his opinion. This explanation was mainly centered on the need to ensure medication adherence.

## 4. Discussion

The aim of our work was to study religious practices with special attention to Ramadan before and after the onset of BD, to assess the perceptions and behaviors related to not fasting during Ramadan in patients with BD and their families’ attitudes, to assess religiosity and self-stigma and their relationships with religious practices, and to explore the doctor-patient relationship around fasting.

In our sample, we noticed that after the onset of BD, some of the patients stopped observing some religious activities such as praying and going to the mosque. This occurrence could be due to residual or subsyndromal symptoms experienced during the interepisodic phases (31) such as lack of interest in work/activities, psychic anxiety, depressed mood and feelings of guilt. These symptoms can explain the changes observed in BD patients’ behaviors related to religious activities as they are known to be associated with impaired social functioning (32).

On the other hand, other patients in our sample reported that they started praying or visiting the mosque after the onset of BD. This finding could be due to the fact that religious coping, as shown by a number of studies, is one of the attitudes that individuals frequently adopt to cope with challenging situations; it has also been proven that this coping method was associated with decreased levels of anxiety and depression as well as an improvement in mental well-being (33). Findings also suggested that positive religious coping may lead to lower self-stigmatization in individuals with BD (34).

In our study, almost two-thirds of the patients fasted regularly before the start of BD. Our result does not correspond with those of a previous report (10) considering 25 Moroccan individuals with bipolar disorder, which reported that almost the entire sample fasted regularly. This could be explained by the small sample size as well as by the difference in the cultural context between Morocco and Tunisia, Tunisia being more secular.

Our results showed that more than half of the patients who fasted regularly before the onset of BD had stopped this practice after BD onset mainly to ensure therapeutic compliance. Some studies have shown how difficult it can be for patients with BD to maintain a normal religious life due to the variability of their disease (35). They have highlighted how difficult it is to maintain institutional religious ties when others might not understand their symptoms or when their treatment regimens come into conflict with their religious beliefs (36).

Our work has also shown that most of the patients adjusted the administration of treatment to suit their fasting; unfortunately, the literature is also lacking on this subject. In fact, we only found two studies which have explored the possible side effects of lithium following the Ramadan fast, which reported contradicting results. One study concluded that the use of lithium during the month of Ramadan did not cause more harmful or toxic effects (37). The only side effects that differed between pre-Ramadan and mid-Ramadan was weight loss, which could rather be explained by fasting than by the effect of lithium, and increased restlessness, which could also be explained by a metabolic disturbance such as hypoglycemia. The other study (8), however, reported an increase in side effects such as feeling thirsty, dry mouth and trembling during the month of Ramadan. Neither study reported significant changes in serum lithium concentrations during Ramadan. However, it should be pointed out that the mean lithium dose was relatively low. Patients needing higher doses or resident in countries where temperatures during the summer can be much higher may develop more side effects (38).

Our results found that the families of most patients who did not fast accepted their decision. There is no literature focusing directly on perceptions and behaviors related to fasting during Ramadan in patients with BD. However, some studies explained how the familial gatherings during periods of religious rituals might offer a greater acceptance and integration of these patients within their family and society (39, 40).

Our results also showed high self-stigma scores in slightly more than a tenth of patients. Self-stigma was significantly associated with negative perception of not fasting, negative remarks regarding not fasting and taking both meals at regular times during Ramadan.

Ramadan is a special time for Muslims, their families and community. Indeed, this month is the occasion for many convivial meetings with the family, for having closer ties with neighbors and friends, notably during the Iftar. Concomitantly, it allows for contact with the wider community and to live spiritual experiences during prayers or visits to the mosque. It is therefore understandable that someone who does not participate in this practice at the same time as the rest of their community may feel left out, excluded from the social group and therefore stigmatized. According to an online survey conducted by TGM Research (41) aiming to understand the attitudes and behaviors of Muslims during Ramadan 2023, and which was conducted in multiple countries, including Tunisia (n = 482), 93% of the sample said that Ramadan is the most awaited season of the Year and 92.3% reported that they will celebrate Ramadan this year.

The participants reported a change in their attitudes during Ramadan. In fact, 56% reported an increase in worship, 58% said that they plan to wake up for suhoor meal, 69% that they will be giving to charity and helping those in need, and 69% plan to attend gatherings with family and relatives. Such important attention accorded by the majority of the sample to this holy month, can explain how the feeling of being marginalized and excluded from society can be accentuated for those who are not taking part in this religious practice.

The literature on self-stigma in BD is sparse. Nevertheless, it suggests that there is a moderate to a high degree of internalized stigma in people with BD (20). Our results remain slightly inferior to those found by the GAMIAN-Europe (Global Alliance of Mental Illness Advocacy Networks) study on experienced discrimination and self-stigma in depression and BD, which showed moderate to high self-stigma scores in more than a fifth of the cases (20, 42).

We also found that the decision whether to fast or not was taken without seeking medical advice in the majority of the sample. Only a small proportion of patients reported that their psychiatrist had spontaneously approached the issue of Ramadan fasting, with advice to not fast in half of the cases. The explanation for such advice was mainly focused on the need to ensure medication adherence.

In the literature, we did not find any work enabling a comparison of the results related to the doctor-patient relationship around fasting. However, a study conducted among patients with diabetes (43) showed that the doctor’s message about fasting in the month of Ramadan was not always present, which was similar to our results although they are two different pathologies.

The majority of our sample stated having decided alone or after talking to an imam about whether to fast or not. The imam as a religious leader holds a significant and influential role in our culture. He is often solicited for important decisions and guidance, serves as a trusted confidant, and asked to provide emotional support and spiritual counseling during times of distress. Considering the role they play and their involvement with people suffering from mental illnesses, we should raise awareness among religious leaders about mental health and view them as allies thus promoting a holistic approach to mental well being.

### 4.1. Limitations

The present study is, to the best of our knowledge, the first to explore the subject of BD and the month of Ramadan in the Tunisian population and the first to specifically study the perceptions and behaviors of patients with BD in relation to their fasting practice during the month of Ramadan.

Due to the paucity of data regarding the perceptions and behaviors of patients with BD in relation to the month of Ramadan and the lack of pre-established standardized measures on which we can rely, this study was above all exploratory and descriptive, and had to include a large number of semi-structured questions and qualitative variables, which should be refined in subsequent studies. In addition, the retrospective collection of certain data may have led to memory bias; the choice of this method was due to the fact that the month of Ramadan only occurs once a year and that certain questions relating to the behaviors and perceptions of patients and their families during this month can only be assessed afterwards. Even though we have tried to include patients with BD of different severity and different socio-economic environment (patients who have been hospitalized or not, patients consulting in the private or public sector), our sample cannot be considered as representative of the entire population of patients with BD. In fact, the majority of the participants were treated at Razi Hospital, which can lead to selection bias since these patients often have more severe clinical forms of the disease. Nonetheless, this study provides a useful initial indication of experiences amongst this population group of interest, which can guide future work in this area.

Moreover, a larger sample size could have allowed for further inferential analyses of these data, which could for now only be explored via descriptive means.

We have also to point out that the “Brief ISMI” scale used in this work had been translated and adapted but not yet validated in Tunisian Arabic dialect.

## 5. Conclusion

Our results showed that almost a third of our patients observed the ritual of Ramadan fasting. This subject seems to therefore be an important matter to which psychiatrists should pay attention in their clinical practice. It is indeed important to respect and validate the concerns of patients regarding their religiosity and to include this aspect in the bio-psycho-social approach. Counseling patients about fasting remains a dilemma for psychiatrists, especially since there is a dearth of literature on this subject and that the few results found were contradictory. This lack of information makes it difficult to develop a clear consensus allowing physicians to have a unified response.

Our results remain exploratory and encourage further work on the role of the environment and social rhythm during Ramadan in patients with BD. Future research should explore these questions with larger samples using appropriate inferential analysis techniques.

Furthermore, it might be interesting to include the challenges linked to the month of Ramadan in psychoeducation programs. The ultimate aim is to optimize the medical care of patients with BD, which requires providing answers regarding the possibility of fasting, reducing the risk of self-stigma and developing targeted therapies in order to improve the prevention of relapses.

## Data availability statement

The raw data supporting the conclusions of this article will be made available by the authors, without undue reservation.

## Ethics statement

The studies involving humans were approved by Razi Hospital Ethics committee (reference: RPA2/2018). The studies were conducted in accordance with the local legislation and institutional requirements. The participants provided their written informed consent to participate in this study.

## Author contributions

IM: Conceptualization, Formal analysis, Investigation, Writing – original draft. UO: Conceptualization, Methodology, Supervision, Writing – review & editing. PG: Supervision, Writing – review & editing. YZ: Investigation, Writing – review & editing. AO: Investigation, Writing – review & editing. FN: Conceptualization, Supervision, Writing – review & editing.

## Abbreviations

BD: Bipolar disorder
HDRS: The Hamilton depression rating scale
YMRS: The young mania rating scale
Brief ISMI: The brief version of the internalized stigma of mental illness scale
